# Strawberry skull in Edwards syndrome

**DOI:** 10.1259/bjrcr.20170045

**Published:** 2017-06-10

**Authors:** Umesh Shriniwas Mudaliyar, Sneha Umesh Mudaliyar

**Affiliations:** ^1^Head of Department Of Radiology, Bhaktivedanta hospital and Research Institute, Mumbai, India; ^2^Pediatric Dentist,Department of Dentistry, Bhaktivedanta Hospital And Research Institute, Mumbai, India

## Abstract

The trisomy 18 syndrome, also known as Edwards syndrome, is the second most common disorder after trisomy 21 (Down's syndrome). It is a chromosomal disorder due to the presence of an extra chromosome 18, which could be either full, mosaic trisomy or partial trisomy 18q. The live born prevalence is estimated as 1/3,000 –1/8,000, but the overall prevalence is higher 1/2500–1/2600 due to the high frequency of foetal loss and pregnancy termination after the prenatal diagnosis. We are presenting a case of Edwards syndrome diagnosed during routine antenatal scan.

## Background

Trisomy 18 syndrome, also known as Edwards syndrome, is the second most common autosomal chromosomal disorder after trisomy 21. It is due to the presence of an extra chromosome 18. The first reported infants were independently described in 1960 by Dr John Hilton Edwards, a British medical geneticist^1^ and Smith et al.^2^ The syndrome pattern comprises a various recognizable pattern of major and minor anomalies, significant psychomotor and cognitive disability associated with infant and neonatal mortality and morbidity.

Trisomy 18 foetuses have numerous major structural anomalies, the highest when compared with trisomy 13 and trisomy 21. The prominent features are: congenital heart disease: 90–95%; central nervous system/spinal abnormalities: 70%; single umbilical artery: 80%; clenched hands with overlap of 2nd and 3rd digits: 80%; rocker bottom feet; horseshoe kidney: 20%, bowel containing omphalocoele: 20–25 and strawberry skull: 43%.^3,4^

## Clinical presentation

A 26-year-old female with no known history of medical or surgical illness conceived for first time after non-consanguineous marriage. She presented herself initially for first trimester nuchal translucency scan at 12 weeks, which did not reveal any clinical abnormality apart from single umbilical artery. However, triple marker test done at 14 weeks revealed marginal increased risk for chromosomal trisomy 18. Patient was not followed up between 14 and 20 weeks. Patient presented herself thereafter for anomaly scan at 21 weeks which revealed structural malformation in foetus as follows.

**Figure 1.**
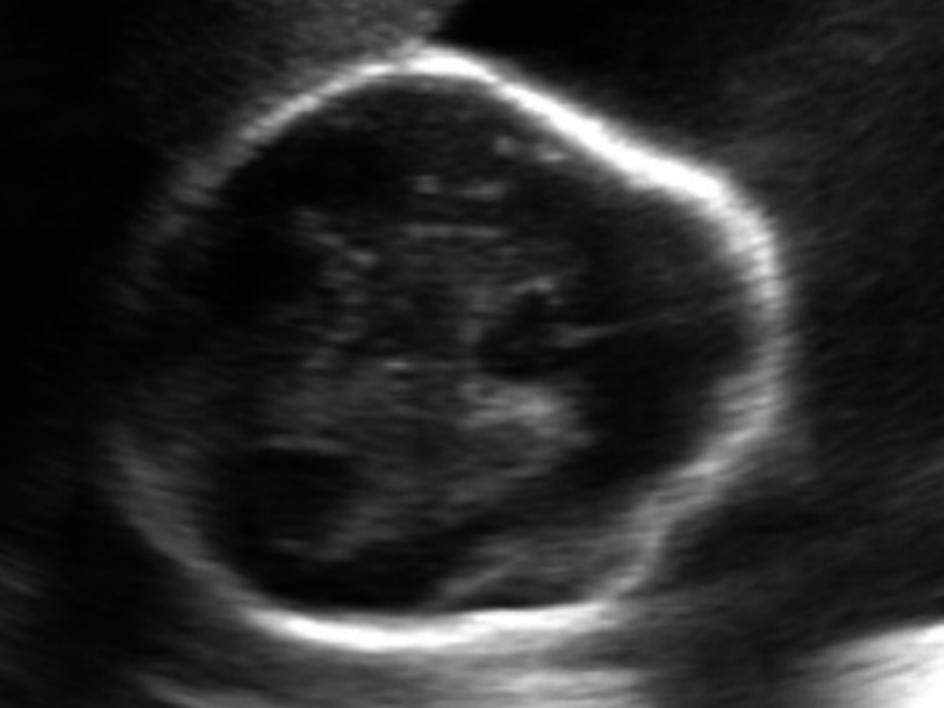
Strawberry skull.

**Figure 2.**
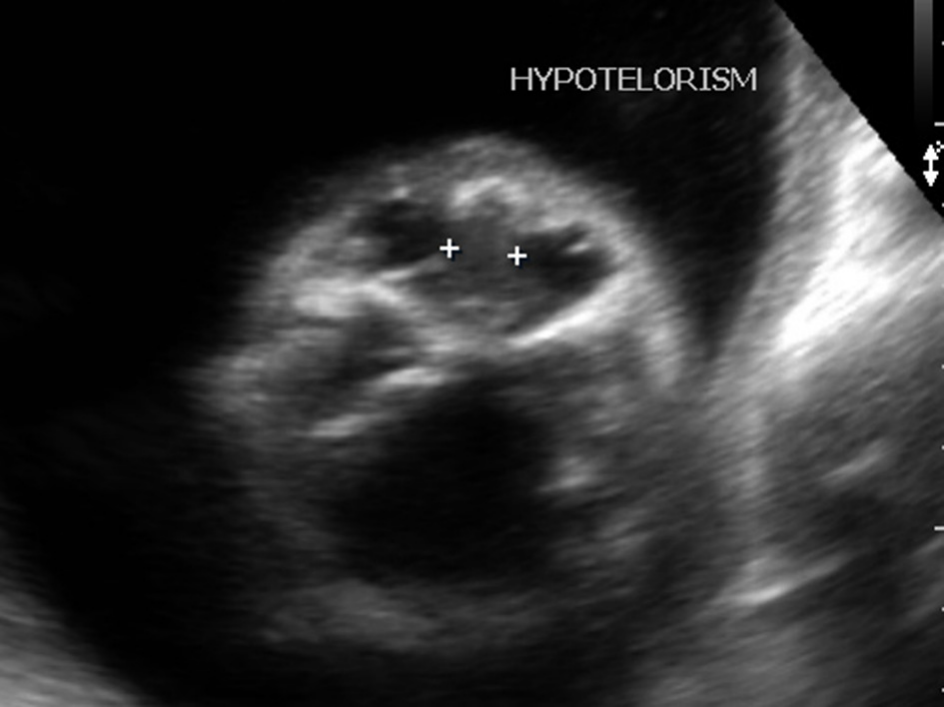
Hypotelorism.

**Figure 3.**
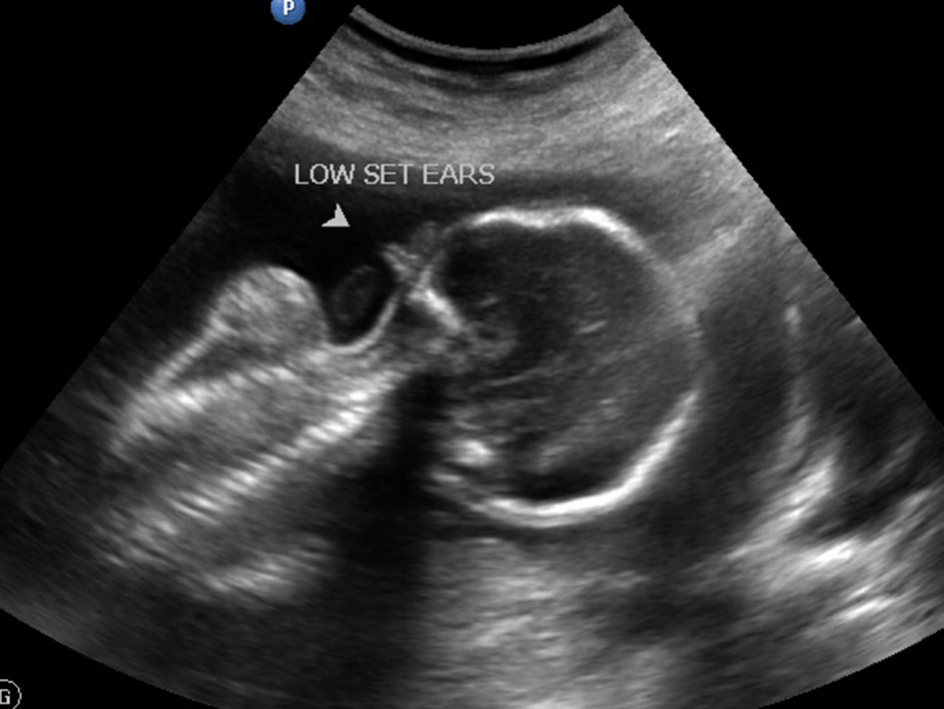
Low set ears.

**Figure 4.**
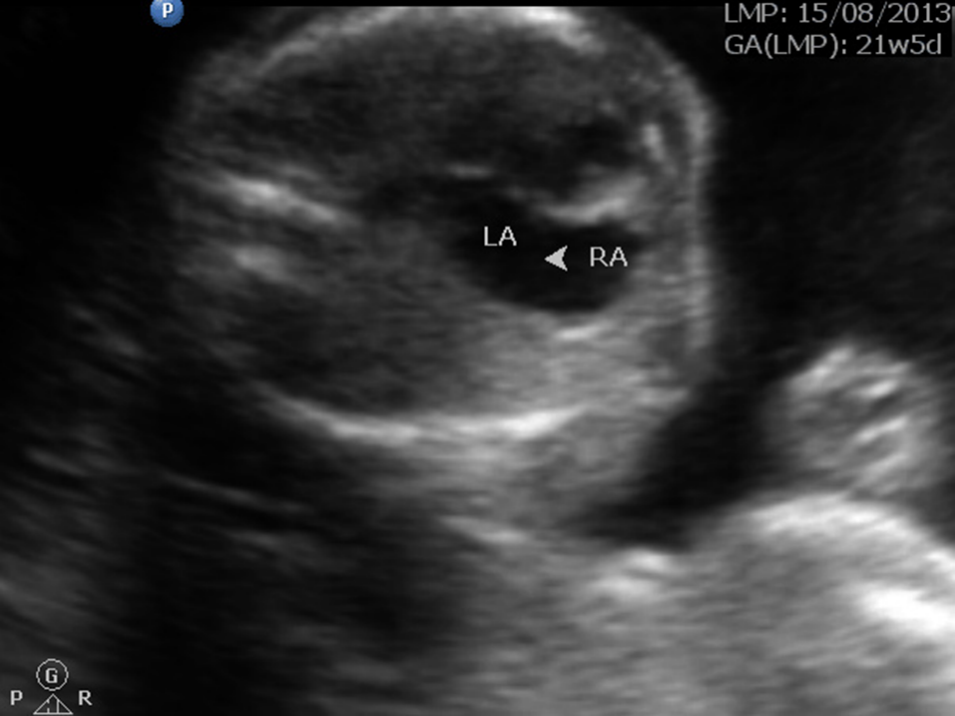
Atrial septal defect.

**Figure 5.**
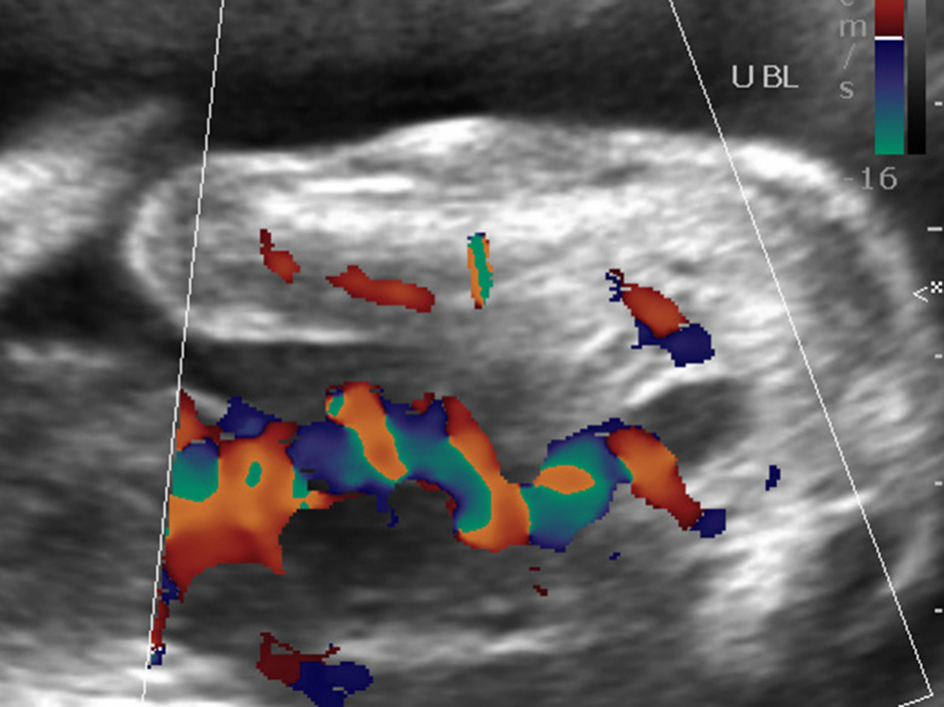
Single umbilical artery seen on axial section.

1. Strawberry shaped skull (Figure 1)
2. Hypotelorism (Figure 2)
3. Low set ears (Figure 3)
4. Wide atrial septal defect (Figure 4)
5. Single umbilical artery with two vessel cord (Figure 5)

The findings apart from the above mentioned images were polyhydramnios and persistent clenched fingers.

## Differential diagnosis

Pseudo-trisomy18/Pena-Shokeir syndrome, an autosomal recessive condition that can contain some overlap in the clinical features of that of trisomy 18.

## Outcome

Patient underwent amniocentesis and foetal karyotyping which confirmed the ultrasound diagnosis Edwards syndrome. After counselling by obstetrician, patient chose for medical termination of pregnancy.

## Discussion

Edwards syndrome is a chromosomal anomaly (trisomy 18). With advances now in banding techniques, it has become easier to differentiate between chromosome 17 and 18. The overall incidence is estimated to be at 1:3000–8000. Karunakaran and Pai^5^ reported the first case in the Indian literature in 1967. Balkrishnan et al in 1971^6^ reported two cases of trisomy E with a particular reference to the overlapping clinical features in Patau's syndrome (Trisomy D) and Edwards syndrome (Trisomy E).

In 1992, Nicholas and colleagues described a characteristic prenatal ultrasound finding of strawberry shaped skull (flat occiput and pointing of frontal bones) in 54 foetuses where the most common anomaly detected was trisomy 18 (in 43 foetuses).^7^

In case of Edwards syndrome, variation in the sex ratio (M/F) from that in the general population was noted in 1962 by Ferguson and Smith.^8^ There is a female preponderance in this condition with a ratio of female to male being 3:1.^8^

The general features constitute a low birth-weight baby with a small placenta, with poor foetal movements and polyhydramnios.

Soft markers such as choroid plexus cysts, delayed growth, nuchal fold thickening, echogenic cardiac focus, echogenic bowel, clenched fingers, rocker bottom feet and pyelectasis are some of the non-specific findings often seen with trisomy 18 and other aneuploidies.

The prognosis of this condition is poor. Majority die *in utero*, even if they survive the natural history is one of limited survival. Eighty percent die in the first month, 50% die by 2 months, 90% by 1 year and remaining 10% are mentally retarded.^9^

Majority of the trisomy 18 cases are cases of full trisomy. It can be non-disjunction as seen in older maternal age groups, the average being 32 years or translocation as seen in young mothers who must undergo chromosomal studies to detect balanced translocation carrier. Patients with balanced translocation have high risk of recurrence in future off-springs.

Early prenatal diagnosis allows the option for safe medical termination of pregnancy. The pregnancies which are carried till term mostly result either into still born babies or newborns with serious congenital defects and postnatal complications. Hence earliest detection of this chromosomal anomaly helps in efficient management of pregnancy as per available alternatives to the expectant mother.

Antenatal foetal ultrasound is a time-tested screening technique for diagnosis of foetal genetic syndromes and other anomalies. The constant ongoing advances in ultrasound technique are aiding in better resolution. This results in increased accuracy in diagnosis in combination with other non-invasive modalities of foetal testing. Thus, the need for invasive foetal testing is reducing.

## Learning points

1. Even though initial 11–13 weeks scan may not warrant significant congenital foetal abnormality, a good structural malformations scan can help to clinch the diagnosis. Active foetal surveillance can help in identifying multiple structural abnormalities which can narrow down the list of differential diagnosis. The most common ultrasound findings are persistently clenched foetal fingers, strawberry skull, single umbilical artery, cardiac defects, intra uterine growth restriction and abnormalities of amniotic fluid.
2. It is important to do active foetal surveillance to look for additional malformations,if a single clinically significant structural defect is identified irrespective of earlier biochemical marker results.

## Consent

Written informed consent for the case to be published (including images, case history and data) was obtained from the patient(s) for publication of this case report, including accompanying images.
